# A Novel Approach for Quantifying Cancer Cells Showing Hybrid Epithelial/Mesenchymal States in Large Series of Tissue Samples: Towards a New Prognostic Marker

**DOI:** 10.3390/cancers12040906

**Published:** 2020-04-08

**Authors:** Louis Godin, Cédric Balsat, Yves-Rémi Van Eycke, Justine Allard, Claire Royer, Myriam Remmelink, Ievgenia Pastushenko, Nicky D’Haene, Cédric Blanpain, Isabelle Salmon, Sandrine Rorive, Christine Decaestecker

**Affiliations:** 1 Department of Pathology, Erasme Hospital, Université Libre de Bruxelles (ULB), Route de Lennik 808, 1070 Brussels, Belgium; louis.godin@ulb.ac.be (L.G.); Claire.Royer-Chardon@erasme.ulb.ac.be (C.R.); Myriam.Remmelink@erasme.ulb.ac.be (M.R.); Nicky.D.Haene@erasme.ulb.ac.be (N.D.); isabelle.salmon@erasme.ulb.ac.be (I.S.); sandrine.rorive@erasme.ulb.ac.be (S.R.); 2 DIAPath, Center for Microscopy and Molecular Imaging, Université Libre de Bruxelles (ULB), CPI 305/1, Rue Adrienne Bolland, 8, 6041 Gosselies, Belgium; cedric.balsat@ulb.ac.be (C.B.); yveycke@ulb.ac.be (Y.-R.V.E.); allard.justine@ulb.ac.be (J.A.); 3 Laboratories of Image Synthesis and Analysis (LISA), Université Libre de Bruxelles (ULB), CPI 165/57, Avenue Franklin Roosevelt 50, 1050 Brussels, Belgium; 4 Laboratory of Stem Cells and Cancer, Université Libre de Bruxelles (ULB), Route de Lennik 808, 1070 Brussels, Belgium; ievgenia.pastushenko@ulb.ac.be (I.P.); cedric.blanpain@ulb.ac.be (C.B.); 5 CurePath, “Centre Universitaire inter Régional d’expertise en Anatomie Pathologique Hospitalière” (CHIREC, CHU Tivoli, ULB), Rue de Borfilet 12A, 6040 Jumet, Belgium

**Keywords:** computational pathology, hybrid E/M state, partial EMT, prognosis, quantification, sequential immunohistochemistry, tissue-based biomarker, urothelial carcinoma, whole-slide imaging

## Abstract

In cancer biology, epithelial-to-mesenchymal transition (EMT) is associated with tumorigenesis, stemness, invasion, metastasis, and resistance to therapy. Evidence of co-expression of epithelial and mesenchymal markers suggests that EMT should be a stepwise process with distinct intermediate states rather than a binary switch. In the present study, we propose a morphological approach that enables the detection and quantification of cancer cells with hybrid E/M states, i.e., which combine partially epithelial (E) and partially mesenchymal (M) states. This approach is based on a sequential immunohistochemistry technique performed on the same tissue section, the digitization of whole slides, and image processing. The aim is to extract quantitative indicators able to quantify the presence of hybrid E/M states in large series of human cancer samples and to analyze their relationship with cancer aggressiveness. As a proof of concept, we applied our methodology to a series of about a hundred urothelial carcinomas and demonstrated that the presence of cancer cells with hybrid E/M phenotypes at the time of diagnosis is strongly associated with a poor prognostic value, independently of standard clinicopathological features. Although validation on a larger case series and other cancer types is required, our data support the hybrid E/M score as a promising prognostic biomarker for carcinoma patients.

## 1. Introduction

In the context of carcinoma pathogenesis and progression, the epithelial-to-mesenchymal transition (EMT) program can be regulated by many signaling pathways and transcription factors, as well as post-transcriptional, epigenetic and post-translational mechanisms [1,2,3]. An epithelial cell is usually considered to have undergone an EMT program if it shows loss of epithelial markers along with increased expression of mesenchymal markers. Key lost epithelial markers include E-cadherin (E-cad, also labeled CDH1), Mucin-1, several cytokeratins (e.g., CK20, CK19, CK18, and CK8), occludin and desmoplakin. Oppositely, N-cadherin, vimentin (VIM), smooth muscle actin, fibronectin, and vitronectin are mesenchymal markers acquired in the course of the transition. Indeed, it has been shown that the EMT program can be manifested in cells to various degrees, meaning that cells may transit through a series of states across the epithelial–mesenchymal spectrum. This suggests that carcinoma cells might adopt and reside in multiple intermediate phenotypic states and these states might be transmitted through multiple cell generations [4]. Progression through the EMT is associated with the acquisition of tumor-initiating potential. Furthermore, the transitions between the epithelial, hybrid E/M, and mesenchymal states are directly related to a cancer cell’s ability to perform the metastatic cascade. Increasing evidence shows that tumor progression and metastasis is favored by carcinoma cell residence in hybrid, partially epithelial, and partially mesenchymal states [2,3,4,5,6,7]. At the metastatic site, cancer cells may reverse their phenotype to regain their epithelial characteristics (using the reverse mesenchymal-to-epithelial transition, MET, process) and form secondary tumors. The EMT program thus gives cancer cells a plasticity that equips them with more aggressive phenotypes, such as tumor-initiating potential and therapeutic resistance. It should be noted that cells that have undergone only partial EMT, giving them a hybrid E/M state combining both epithelial and mesenchymal features, are more likely to acquire stem-like properties. Partial EMT and hybrid E/M cancer cells thus represent a promising target for treatment of cancer [2,3]. Consequently, characterization of the hybrid E/M status of tumors could provide useful biomarkers for diagnostic, prognostic, and theranostic purposes.

The EMT-related data summarized above have mainly been derived from in vitro cell culture models leading to frequent debates as to whether EMT and partial EMT are truly relevant to cancer in vivo [1]. A recent study has identified several subpopulations of tumor cells showing different partial EMT degrees in animal models [4]. These different subpopulations are functionally distinct and have different clonogenic potentials, invasive properties, differentiation abilities, and plasticity phenotypes. The different intermediate E/M states are associated with a specific transcriptional signature and are located in different regions and microenvironments within tumors.

Co-expression of epithelial and mesenchymal markers has also been evidenced in human cancer tissue samples, using different methodical approaches including immunofluorescence (IF), DepArray, and CyToF [8,9,10,11]. However, to the best of our knowledge, this co-expression was established for only a few cases, with a maximum of 18 cases in Navas et al. [12] using an IF assay. In this latter study, a method was proposed to quantitate the individual and colocalized expression of E-cad and VIM. Previously, Polioudaki et al. [13] also proposed a quantitative IF approach to characterize the E/M status of circulating tumor cells by assessing keratin and VIM expression levels. However, quantification was only carried out on five patients.

To date, studies analyzed the prognosis value associated with EMT-related biomarkers in human cancer tissue [8,9,14,15,16]. However, these studies did not assess the hybrid E/M status of cancer cells. This requires co-expression analysis at cell level on large sample series. In addition, several authors have proposed EMT gene-related signatures extracted from human tumors [17,18,19,20,21,22]. These signatures result from global expression profiling data, representing the aggregate of the various cell populations within the sample (including immune and other stromal cells). Interestingly, Georges et al. [22] have developed a logistic regression model aiming to distinguish between the three categories E, M, and E/M, based on two predictors, the E-cad/VIM ratio and Claudin 7 gene product, and 20 other gene products as normalizers. However, this model was established using cell lines previously categorized using the E-cad/VIM ratio (but based on protein expression) and validated at single-cell level only in vitro. Cell-sorting techniques can further be used to improve the specificity of genomic signatures and can be combined with single-cell sequencing to characterize the hybrid E/M status of cancer cells [23]. However, these sophisticated techniques are often costly, difficult to transpose into clinical practice and therefore remain a research tool.

All these constraints motivated us to develop an alternative approach allowing the identification and quantification of cancer cells with a hybrid E/M phenotype in large series of tumor samples. Our approach is based on sequential chromogenic immunohistochemical multiplex (SCIM) which consists of a sequential process being applied multiple times on the same tissue slide. The process itself involves standard immunohistochemistry (IHC) staining and slide scanning, followed by color washing before continuing with a new cycle. Finally, image processing is used to realign the virtual slides so produced and to detect and quantify staining colocalization. This approach allows the detection of proteins co-expressed in a cell with the same subcellular location, bypassing the problem of chromogen superposition, and unlike IF approaches, ensures easy morphological characterization and identification (i.e., direct access to location of IHC staining at the histological, cellular, and subcellular levels). In the present study we implemented SCIM to identify and quantify cancer cells co-expressing VIM and pan-cytokeratin (pan-CK), which allowed us to evidence and quantify the presence of cells with a hybrid E/M state in whole carcinoma slide images. We chose to use pan-CK and VIM as biomarkers rather than E-cad and VIM (as done in some previous studies, see above), because a loss of E-cad expression has been reported as potentially being an early event in the progression of EMT [24,25], as we observed in the case of urothelial carcinomas (see Results). The choice of pan-CK thus ensured easier identification of a maximum of tumor cells before they eventually switched to the mesenchymal state. We were able to validate our methodology on small series of lung and urothelial carcinomas and to investigate the potential prognostic value of our hybrid E/M score on an extended series of urothelial carcinomas. Results obtained so far have been very encouraging and demonstrate the clinical interest in detecting such hybrid E/M cancer cells in histological samples.

## 2. Materials and Methods

### 2.1. Material

Formalin-fixed and paraffin-embedded (FFPE) tissue samples of human cancers were provided by the Biobank of the Pathology Department of the Erasme hospital after ethical committee agreement (P2017/578). A series of 26 lung and 31 urothelial carcinomas was used to validate the methodology. The urothelial series was further extended to a total count of 113 primary urothelial carcinomas to investigate the prognostic value of the hybrid E/M score defined below. To this end, a number of different clinicopathological features were collected: patients’ age, gender, adjuvant treatment, disease-free survival, and overall survival. Anatomopathological data included grade according to WHO 2016 [26], presence of a divergent differentiation, lymphovascular invasion, multifocality, presence of concomitant in situ carcinoma lesions, and pTNM stage according to UICC 2017. All urothelial tumors were from patients not previously treated (primary tumor resection). Histopathological diagnoses were reviewed and characterized by a uropathologist. Data distributions are detailed in the Results section.

### 2.2. Sequential Chromogenic Immunohistochemical Multiplex (SCIM) to Evidence VIM/Pan-CK Co-Expression

**Figure 1:**
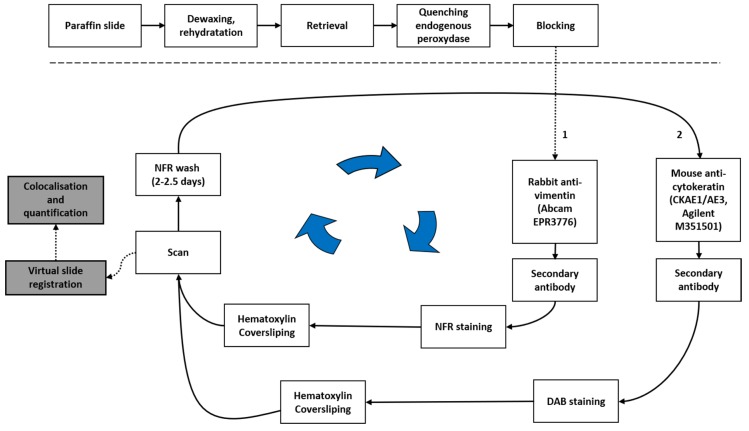
Sequential chromogenic immunohistochemical multiplex (SCIM) to evidence cells co-expressing vimentin (VIM) and pan-cytokeratin (pan-CK). See the main text for details. NFR: napthol and Fast Red; DAB: diaminobenzidine.

Figure 1 outlines the SCIM technique we implemented to evidence cells co-expressing VIM and pan-CK, and is detailed as follows. Tissue sections (5 µm thick) were subjected to standard IHC on a Ventana Discovery XT^TM^ (Ventana Medical Systems, Inc., Tucson, AZ, USA) using the RedMap detection system according to the manufacturer’s protocols. Briefly, the FFPE tissue sections were deparaffinized and rehydrated. Antigen retrieval solution (EDTA, pH8.4, cat. no. 950-124; Ventana Medical Systems, Inc.) was applied for 36 min at 100 °C. Then, the slides were incubated with the rabbit monoclonal anti-VIM antibody for 60 min at 37 °C (1:1250, clone EPR3776, cat. no. ab92547; Abcam, Cambridge, UK.). The slides were washed and incubated with the biotinylated anti-rabbit secondary antibody for 24 min (1:200, cat. no. BA-1000, Vector Laboratories, Ltd., Peterborough, UK) followed by the addition of complex streptavidin-alkaline phosphatase. Immunostaining was detected by incubation with naphthol and Fast Red (NFR). The IHC slides were counterstained with Gill’s hematoxylin for 2 min at room temperature, dehydrated, and mounted. The whole slides were digitized at 20× (0.453 µm side pixel) using a NanoZoomer 2.0 HT scanner (Hamamatsu, Hamamatsu city, Japan), which was previously calibrated using a specific slide provided by the manufacturer. The IHC slides were then demounted, incubated in 100% ethanol for two days, until complete red color erasing, and rehydrated. The slides were then submitted to a second IHC protocol to evidence pan-CK expression. The slides were incubated with a mouse monoclonal anti-pan-CK antibody for 60 min at 37 °C (1:200, clone CKAE1/AE3, cat. no. M351529, Agilent Technologies Belgium S.A./N.V., Diegem, Belgium). The slides were then washed and incubated with the biotinylated anti-mouse secondary antibody for 24 min (1:200, cat. no. BA-2001, Vector Laboratories, Ltd.) followed by the addition of complex streptavidin-horseradish peroxidase. Pan-CK immunostaining was detected by incubation with diaminobenzidine (DAB) and hydrogen peroxide. Finally, the IHC slides were counterstained with Gill’s hematoxylin for 2 min at room temperature, dehydrated, mounted, and digitized (as mentioned above for VIM).

To control the epithelial vs. hybrid E/M status of carcinoma cells, E-cad expression was evidenced on a tumor section consecutive to a pan-CK one. We used a mouse monoclonal anti-E-cad antibody incubated for 2 hours at room temperature (1:50, clone NCH-38, cat no. M361201-2, Agilent) and the same anti-mouse secondary antibody mentioned above was added but this time incubated for 60 min at 1:100 dilution. After incubation with DAB and hydrogen peroxide, the slides were counterstained with Gill’s hematoxylin for 2 min at room temperature, dehydrated, mounted and digitized.

### 2.3. Image Processing and Hybrid E/M Scoring

First of all, the whole-slide images were normalized using a methodology that we previously detailed and validated [27]. This approach ensured that the quantitative IHC analyses were not impacted by possible staining variations between the different IHC batches, which were required to process the large slide series.

**Figure 2:**
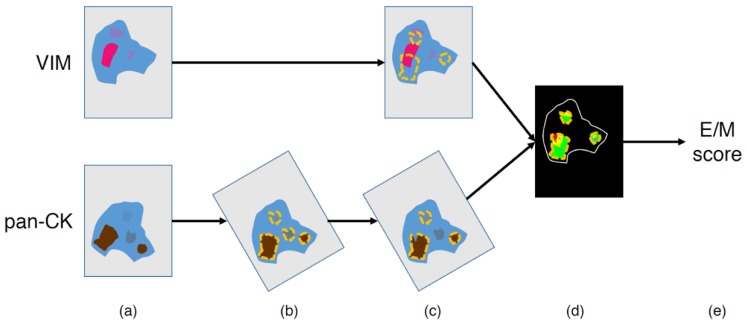
Image processing workflow to quantify the hybrid epithelial/mesenchymal (E/M) score. (**a**) Input images, (**b**) image registration and automatic annotation of the pan-CK-positive (pan-CK+) regions at low (4×) magnification, (**c**) manual exclusion of irrelevant annotations (e.g., necrosis areas and/or tissue defects), (**d**) Vim-positive (VIM+) and pan-CK+ detection in the final annotations (cf. details in Figure 3), and (**e**) E/M score computation (cf. details in Figure 3).

After this normalization step, image processing and analysis were performed using Visiomorph DP 2017.4 (Visiopharm, Hoersholm, Denmark) to determine VIM and pan-CK co-expression in each tissue slide. For this purpose, each pair of virtual VIM and pan-CK slides, obtained after applying SCIM to the same tissue slide, was subjected to image registration using the TissueAlign add-on module (see Figure 2a,b). Registration accuracy was evaluated using control points manually placed by an expert on the images to align, as previously described [28]. Briefly, the accuracy of the registration was measured at seven different locations uniformly distributed on each of 57 virtual slide pairs (from 26 lung and 31 urothelial carcinomas) and then averaged per slide pair.

The pan-CK-positive (pan-CK+) and VIM-positive (VIM+) areas were then automatically detected in the aligned virtual slides to evidence their co-expression in tumor cells. The process used Visiopharm’s color deconvolution to identify the channels corresponding to DAB, FastRed, and HEM, where low values correspond to dark staining. The first step consisted of a fast and automatic annotation of the pan-CK+ regions carried out at 4× magnification (2.265 µm-side pixel) on the virtual pan-CK slide: after thresholding the pixel values in the deconvoluted DAB channel, the smallest objects and smallest holes (<100 µm^2^) were respectively removed and filled, and the region of interest was eroded with a 3 by 3 pixels mask. This eroding step helps to avoid false detection of pan-CK and VIM co-expression in the next step of the processing, in case of strong proximity between pan-CK+ and VIM+ staining (e.g., in case of immune infiltrates) and/or staining burr. The resulting annotations were then visually controlled and manually corrected by an expert in order to exclude irrelevant parts, such as necrosis or tissue defects (see Figure 2c). It should be noted that due to image registration, the annotations carried out on the virtual pan-CK slide are automatically applicable on the registered virtual VIM slide (Figure 2c). In the regions of interest validated by the expert, the VIM+ and pan-CK+ areas were then automatically detected with greater precision at higher resolution by processing the deconvoluted Fast Red and DAB channel, respectively, (see Figure 2d). We investigated two resolution levels for this step (i.e., 5× and 10× magnification with 1.812 µm- and 0.906 µm-side pixel, respectively,) to take into account registration accuracy, as detailed in the Results section. The method used for this staining detection step is similar to the one used for the automatic annotation of pan-CK+ regions at 4× magnification (see above). It can be noted that prior to applying thresholds, the deconvoluted channels were smoothed using a median filter (3 by 3 mask).

Before computing pan-CK and VIM co-expression areas, cell nuclei, which are negative for each marker, were excluded. This exclusion aims to reduce the impact of an image alignment that would not be absolutely perfect on co-expression measurements. A pixel was considered as belonging to a nucleus if the following two criteria were met: (1) the average of the two deconvoluted HEM values computed on the image pair was sufficiently low (i.e., blue color sufficiently strong) and (2) the pixel was identified as belonging to a round structure using Visiopharm’s Polynomial Blobs-filter. Once the segmentation was done, we excluded any object too small (<4 µm^2^) to be a cell nucleus.

**Figure 3:**
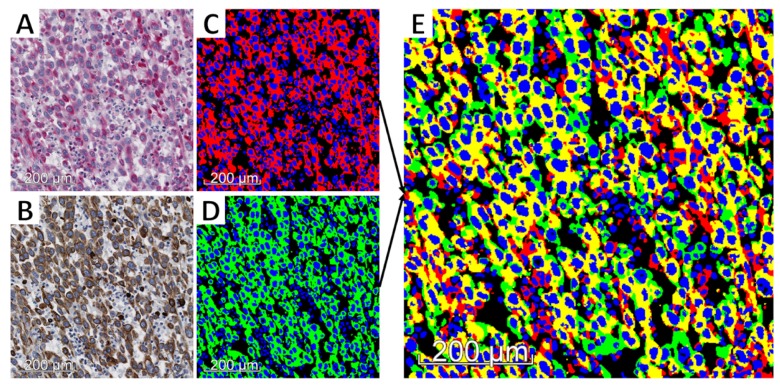
Steps of the hybrid E/M score computation. Registered images (from the same tissue slide) processed at 10× magnification (0.906 µm-side pixel): (**a**,**b**) VIM and pan-CK expression, respectively, on registered image areas; (**c**,**d**) detection of cell nuclei (blue), VIM+ (red) and pan-CK+ (green) areas; and (**e**) detection of pan-CK+/VIM+ area (yellow), pan-CK+/VIM− area (green), pan-CK-/VIM+ area (red). The hybrid E/M score was computed as the ratio between the yellow area and the sum of the yellow and green (i.e., pan-CK+) areas.

Finally, different tumor areas of interest were determined, especially the pan-CK+/VIM− and pan-CK+/VIM+ areas, corresponding to epithelial state and hybrid E/M state, respectively. The hybrid E/M score is defined as the ratio between the pan-CK+/VIM+ area and the whole pan-CK+ area (i.e., the sum of the pan-CK+/VIM− and pan-CK+/VIM+ areas, see Figure 2e and Figure 3). As mentioned above, these measurements were evaluated at two magnification levels (5× and 10×).

**Figure A1:**
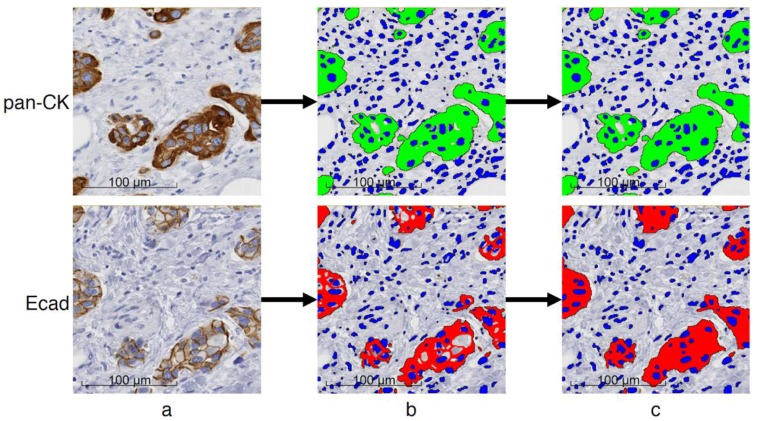
Image processing to quantify cells expressing pan-CK and E-cad on two consecutive slides. (**a**) Input images shown at 20× magnification (0.453 µm-side pixel), (**b**) intermediary result after the thresholding step identifying DAB staining and cell nuclei, and (**c**) final result after both thresholding and post-processing to identify whole cell areas except the nuclei.

In addition, we quantified another feature also related to EMT—the loss of E-cad expression. As E-cad is always co-expressed with pan-CK, we quantified these two markers on virtual serial slides at a 5× magnification, after registration to facilitate expert control and annotations (as described above). As E-cad is mainly expressed in the cell membrane, we aimed to identify the whole surface of cells expressing E-cad in order to be comparable to the quantified pan-CK+ area in epithelial cells (see Appendix Figure A1). E-cad+ (membrane and cytoplasmic) areas were first detected by thresholding the pixel values in the deconvoluted DAB channel, then removing the smallest objects (<4 µm^2^) and applying a morphological closing operation (cf. Appendix Figure A1a,b). To identify whole cell areas in case of membrane staining, we used Visiopharm’s features to select light areas (i.e., intensity > 225) of less than 250 µm^2^ in surface and surrounded by more than 33% of positive areas (corresponding to membrane staining) as the surface area of a cell expressing E-cad (cf. Appendix Figure A1c). As for pan-CK and VIM co-expression detection, cell nuclei were excluded. The E-cad score is defined as the ratio between the total E-cad+ cell area and the total pan-CK+ area. It is expected to be near to 1 in the absence of partial EMT and decrease with loss of E-cad expression, thus being inversely correlated to the hybrid E/M score.

### 2.4. Data Analysis

The statistical analyses were performed using Statistica 7.1 software (StatSoft, Inc., Tulsa, OK, USA). We used the Mann–Whitney test to compare pairs of independent groups of quantitative data and Fisher’s exact test to analyze the associations between binary variables. The relationship between two quantitative variables involved linear (Pearson) or non-parametric (Spearman) correlation analysis. Survival analyses were carried out using Kaplan–Meier analysis, log-rank test, and univariate and multivariate Cox regression method.

## 3. Results

### 3.1. SCIM Validation

**Figure 4:**
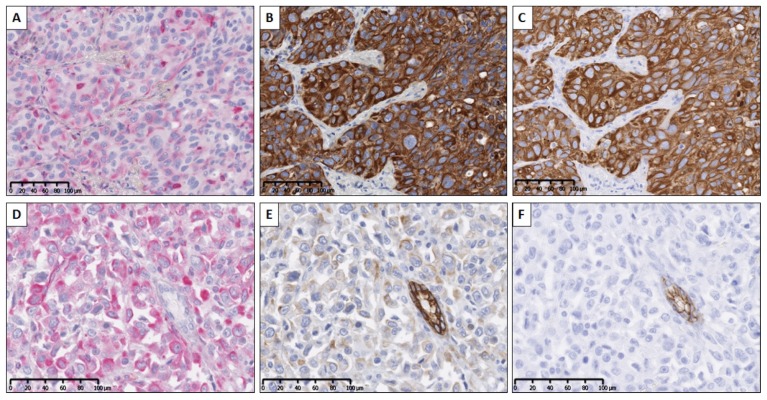
SCIM (**a**–**c**) and hybrid E/M status (**d**–**f**) validation. (**a**) VIM expression evidenced in pink (by using naphthol and Fast Red (NFR)) in a poorly-differentiated lung adenocarcinoma (ADC) tissue section. Pan-CK expression revealed (by means of diaminobenzidine (DAB)), (**b**) the same tissue section, by means of immunohistochemistry (IHC) staining performed after erasing NFR (sequential chromogenic immunohistochemical multiplex (SCIM) technique), and (**c**) a serial tissue section using standard IHC. Using two serial slides from a pleomorphic lung ADC: IHC expression of VIM (**d**) and pan-CK (**e**) on the same tissue section (using SCIM), and E-cad (**f**) on a consecutive tissue section.

Tests were performed in order to validate the SCIM technique. Using primary antibodies from different species no antibody denaturation was required. We first verified the strict specificity of the anti-rabbit secondary antibody for rabbit IgG. For this purpose, slides were incubated with the anti-VIM antibody (made in mouse) and then washed and incubated with the biotinylated anti-rabbit secondary antibody. No staining was observed. The second control consisted in the validation of pan-CK staining in terms of sensibility after erasing NFR (used to reveal VIM expression) with an ethanol bath. For this purpose, the VIM/pan-CK SCIM technique was applied to two samples of lung carcinoma, followed by standard anti-CK IHC staining on a consecutive slide from the same two samples (as illustrated in Figure 4a–c for one of them). This experiment enabled us to check for CK expression pattern similarity between the two staining methods and so confirm antigenicity preservation after NFR erasing.

Finally, we verified the epithelial vs. hybrid E/M status of cells by considering E-cad expression. Figure 4d–f illustrate the staining patterns of the three markers on two serial slides (one for VIM/CK using SCIM and the following for E-cad) from a lung cancer sample. It evidences the colocalization of E-cad expression with strong pan-CK expression in VIM-negative areas, whereas VIM/pan-CK colocalization areas correspond to E-cad-negative areas (and weaker pan-CK expression). In the next section we provide quantitative data which evidence the link between the gain of the E/M status and the loss of E-cad expression in tumor tissue.

### 3.2. Validation of Image Registration and Hybrid E/M Score Evaluation

**Figure 5:**
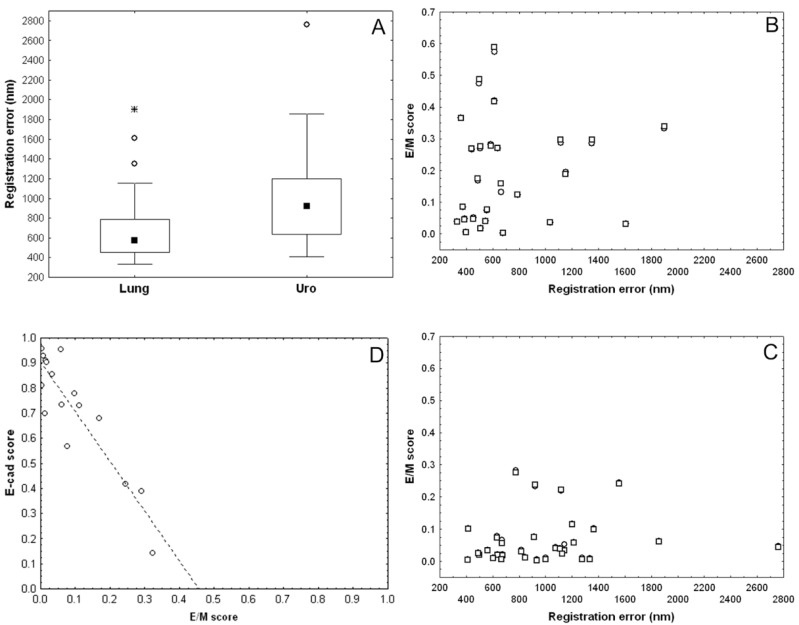
Validation of image registration and hybrid E/M score evaluation. (**a**) Registration errors (nm) on pairs of VIM and pan-CK virtual slides. E/M scores computed on lung (**b**), and urothelial (**c**) carcinoma samples at 10× (dots) and 5× (overlapping squares) magnification levels related to the registration error. Dots are not visible in cases of perfect match between the two scores. (**d**) Negative correlation between E-cad score and E/M score in urothelial carcinomas (Pearson r = −0.90, *n* = 16).

Registration errors were measured on 57 pairs of virtual slides provided by the SCIM technique targeting VIM and pan-CK expression on lung (*n* = 26) and urothelial (*n* = 31) carcinoma samples. On the complete series the average error was 863nm, with lower registration errors for lung samples as compared to urothelial ones as shown in Figure 5a (Mann–Whitney test: *p* = 0.0046). The registration error remained lower than 2 µm (except one outlier), i.e., about the pixel side size at 5× magnification, and was lower than 1 µm (i.e., about the pixel side size at 10×) for more than 75% of lung samples and more than 50% of urothelial ones (Figure 5a). These data motivated us to compare the hybrid E/M scores computed at 10× and 5× magnification. Figure 5b,c show a very good match between the paired E/M scores calculated at 10× resolution (denoted by dots) and those at 5× (denoted by overlapping squares), and extracted from the same slide and thus with the same registration error (x-value). This indicates that there is little to no difference between the two scores. In addition, the absolute differences between scores at 5× and at 10× did not significantly increase with registration error, as indicated by a very low (and not significant) Spearman correlation between these two features (r_s_ = 0.24 with *p* = 0.25 for the lung series, and r_s_ = 0.22 with *p* = 0.24 for the urothelial one). However, the E/M scores were higher in lung tumors than in urothelial tumors, with median scores of 15.3% and 4.6% (at 10× magnification), respectively (Mann–Whitney test: *p* = 0.001).

Finally, we also compared the E/M score and the E-cad score in a series of 16 urothelial carcinomas exhibiting E/M scores that were representative of the range of values observed (Figure 5d). As expected, we observed a strong negative correlation between the two scores (Pearson r = −0.90). The linear trend shows that the E-cad score decreased about twice as fast as the E/M score increased. These preliminary data suggest that E-cad loss could be faster than VIM gain at the early stages of the EMT process in this type of tumor.

### 3.3. Presence of Hybrid E/M Tumor Cells: A Potential Aggressiveness and Prognostic Biomarker for Urothelial Carcinomas

For the complete series of 113 urothelial carcinomas, we quantified the hybrid E/M score at 5× magnification to accelerate the process (by at least a factor 2) without affecting the measurements (see Figure 5c). 

In this series, the average patient’s age is 72 years with a standard deviation of 9.90 years. Ninety-three patients (82.31%) were male. Regarding location, 93 urothelial carcinomas (82.31%) were from the bladder and 20 (17.69%) from the upper urinary tract.

**Figure 6:**
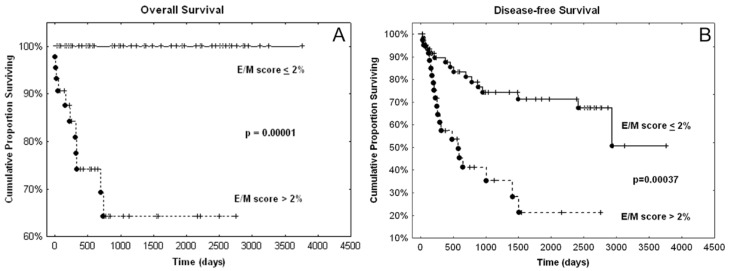
Overall survival (OS) (**A**) and disease-free survival (DFS) (**B**) curves resulting from the binarized E/M score. The dots indicate cases of death (**A**) or recurrence (**B**), while the crosses indicate live (**A**) or disease-free (**B**) patients. *p*-values resulted from log-rank test.

Eleven patients died from their cancer with a median overall survival (OS) of 228 days (about 7.5 months), whereas 36 patients recurred with a median disease-free survival (DFS) of 310 days (about 10 months). We observed that the carcinomas of the deceased patients were all characterized by an E/M score greater than 2%. Figure 6 shows that the E/M score binarized with respect to this threshold has a very significant prognostic value for OS and DFS. In contrast, the quantitative E/M score has no significant prognostic value (univariate Cox analyses: *p* = 0.18 for OS and *p* = 0.51 for DFS). These data suggest that it is not the amount but merely the presence of a minimum of tumor cells in hybrid E/M states that contributes to disease aggressiveness.

Other features confirm the relation between the presence of hybrid E/M cancer cells and disease aggressiveness. Indeed, as detailed in Table 1, the presence of hybrid E/M cells is associated with high stages, high grades, the presence of variant histological differentiation, and concomitant in situ carcinoma (CIS).

Finally, univariate and multivariate survival analysis performed on all the available clinicopathological variables identified the presence of CIS and lymph node metastasis (N+) as the two most contributive prognostic factors for disease recurrence in our series (cf. Appendix Table A1). Table 2 shows that the binarised E/M score significantly contributes as an independent and poor prognostic factor to the Cox model involving these two clinicopathological variables. The fact that this binarized score identified a subgroup in our series where all patients were alive (if hybrid E/M score < 2%, see Figure 6a) prevents the binarized score from being included in a multivariate Cox model focusing on overall survival. Using the quantitative hybrid E/M score instead of the binarized one provides no significant contribution in multivariate models and thus confirms the univariate Cox analyses mentioned above.

The hybrid E/M score we proposed does not take into account staining intensity and could therefore be refined by considering different contributions based on the combinations of weak vs. strong pan-CK staining and weak vs. strong VIM staining. The different combinations thus encountered could be indicative of different stages of partial EMT. In our series, we quantitatively determined such weak and strong expression based on thresholds applied on intensity measurements. In our series of urothelial cancers, we observed that the VIM expression was predominantly weak, whereas the strong and weak pan-CK contributions were better balanced. Consequently, we computed a refined score which quantified the proportions of cancer cells with (weak or strong) VIM expression and weak pan-CK expression, labeled “advanced E/M score”. We first observed a strong correlation (both Pearson r and Spearman r_s_ = 0.97) between the two E/M scores. The advanced E/M score thus provided very similar results to those reported above:

- All the death patients had cancers with an advanced E/M score larger than 1%.
- The advanced E/M score binarized with respect to this threshold had a very significant prognostic value, as evidenced by the log-rank test for OS (*p* = 0.00012) and DFS (*p* = 0.00034).

In fact, only 10 cases (i.e., 9%) showed a discordant status between the two scores, i.e., having E/M scores < 2% and advanced E/M scores > 1%. However, these 10 cases did not differ in terms of prognosis (regarding both OS and DFS) from either the 59 cases with global E/M score < 2% and advanced E/M score < 1% or the 44 cases with global E/M score > 2% and advanced E/M score > 1%. To complete the above results, Table 3 shows that the Cox Model that is obtained when the binarized advanced E/M score replaces the binarized E/M score is very similar to that provided in Table 2. The HR of the advanced score appears slightly higher but belongs to the 95% CI (1.2782, 5.4429) of the global score HR in Table 2.

All these data strongly suggest that the two highly-correlated scores provide very similar prognostic values. However, the advanced E/M score requires that we distinguish weak and strong CK expression, while the (global) E/M score does not and is thus easier to reproduce.

## 4. Discussion

Over the past few years, multiplexing has emerged as a useful tool for identifying several tissue-based biomarkers on the same tissue section. Compared to a classical analysis of different IHC biomarkers evidenced on serial tissue sections, multiplexing provides critical information to better understand and characterize at a cellular level, biological events involved in tumor progression or resistance to treatments [9,29]. The present work aims to identify the cellular co-expression of biomarkers in order to quantify the presence of hybrid E/M cancer cells in a large series of human cancerous tissue samples.

**Figure A2:**
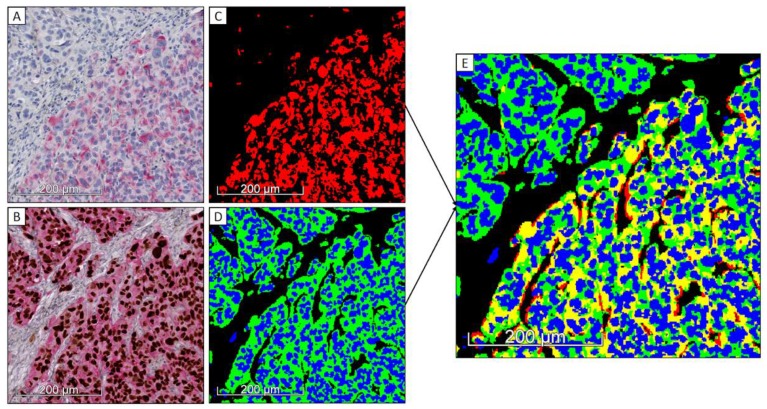
Illustration of the E/M score computation steps adapted for patient-derived xenograft model. Registered images (from the same tissue slide) processed at 10× magnification (0.906 µm-side pixel): (**a**) human-specific VIM expression; (**b**) standard double IHC step for evidencing pan-CK expression (red staining) and Ku80 (nuclear marker for identifying human cells in animal model, brown staining); (**c**,**d**) detection of VIM+ (red) and pan-CK+ (green) areas, and human cell nuclei (blue); (**e**) detection of pan-CK+/VIM+ area (yellow), pan-CK+/VIM− area (green), and pan-CK−/VIM+ area (red). The hybrid E/M score is computed as the ratio between the yellow area and the sum of the yellow and green areas. This methodology was recently applied in a study submitted for publication.

Multiplexing can be carried out using either chromogenic IHC or IF approaches, each one having its own advantages and disadvantages, as reviewed in Parra et al. [29]. IF is often considered more appropriate for co-expression analysis than chromogenic IHC, especially since there is no need to repeat the staining and image acquisition steps (with an intermediary color-erasing step), combined with easier image processing (usually no need of image registration and easier signal detection). However, IF is subject to other technical constraints (such as tissue autofluorescence and fluorochrome bleaching) and requires more expensive equipment and less common technical expertise for tissue processing and image acquisition. Furthermore, additional markers (e.g., targeting cell membranes and nuclei) should be included in the process to allow morphological characterization and identification, and may complicate image interpretation. The resulting constraints are more difficult to accommodate in standard histopathology workflow. This is probably why the number of cases analyzed by IF approaches are often small (as mentioned in the Introduction) and mainly used for illustrative purposes in EMT studies [8,9,10]. On the contrary, the SCIM application we propose allows the analysis of E/M biomarkers co-expression in large series of tissue samples as well as easy morphological scrutiny. Of great interest, this methodology can also be adapted for mouse-model-bearing human tumor cells (i.e., patient-derived xenograft) as presented in the Appendix (Figure A2).

In the present work, we tested our method on urothelial cancer. As mentioned by Garg and Singh [30]), urothelial cancer today requires attentive monitoring, making bladder cancer an expensive disease to manage. Novel markers of aggressiveness are thus needed to help clinicians make their therapeutic decisions with a direct impact on cancer-specific recurrence/death. As also pointed out by Garg and Singh, EMT is a promising area of investigation for risk stratification of pathologically similar urothelial tumors. Urothelial cancer therefore presented itself as a prime candidate to evaluate our SCIM methodology and to assess the prognosis value of partial EMT. The obtained results suggest that our E/M score could be very useful in daily practice to identify urothelial carcinoma that should benefit for a more aggressive treatment in view of the increased risk of recurrence inferred from the presence of hybrid E/M cells.

The literature mentions the occurrence of shifts from membranous to cytoplasmic E-cad staining in tumor buds (i.e., in small clusters of cancer cells) [31]. Whereas there is emerging evidence that these buds present hybrid E/M phenotypes [31,32], our data on urothelial carcinomas rather suggest a loss of E-cad expression, which would be faster than the gain in VIM at the early EMT stages in this type of cancer cell. This faster decline agrees with the fact that the loss of E-cad is considered an early EMT event [24,25]. It can also be noted that tumor budding in urothelial carcinomas seems to be associated with specific molecular patterns, where collagens play significant roles and would be involved in loss of E-cad, as observed by Miyake et al. [33].

However, all the above results need to be confirmed with larger series, in particular to be able to perform stratified analyses regarding the T variable and other clinocopathological variables.

Prognostic value of EMT profiles in urothelial carcinomas has been studied during recent years using either genomic signatures [19] or semi-quantitative analysis of IHC biomarkers [34,35,36]. Directly related to our findings, low expression of E-cad was shown to be an independent prognosis factor of poor progression-free survival in the case of non-muscle invasive (Ta-T1) bladder cancer [34,35]. Similarly, Cho et al. [36] found that loss of E-cad expression and the presence of VIM expression in at least 20% of tumor cells (visually identified) were associated with poor prognosis in a series of 93 upper urinary tract urothelial carcinomas. The present quantitative study suggests that the appearance of a very small percentage of tumor cells expressing VIM is sufficient to confer a poor prognosis. The highly negative correlation we evidenced between the E-cad and E/M scores agrees with the poor prognosis value associated with a loss of E-cad expression. Finally, sarcomatoid urothelial bladder cancers, known for their propensity to develop distant metastases and associated with shorter survival, show EMT network dysregulation compared to conventional invasive bladder cancers [20,37]. It should be noted that our series included five sarcomatoid cancers that all had an E/M score > 2%.

In order to account for different partial EMT stages in a second step, we considered pan-CK staining intensity in the quantification of an advanced E/M score that focuses on cancer cells with VIM and weak pan-CK coexpression. This advanced E/M score is highly correlated with the “global” E/M score and, as expected, provides very similar prognostic value to that of the latter. In contrast to in vitro and animal model studies, clinical series are composed of resected tumors at various and uncontrolled stages of evolution. In this context, our data suggest that it is merely the presence of hybrid E/M cells, not their quantity that provides prognostic value. These results should be validated on larger case series, in particular to determine whether the prognostic value of this type of biomarker can be improved by taking into account a more specific partial EMT stage, such as those associated with either a loss of E-cad expression or a weak pan-CK expression, potentially considered as early and late EMT stages, respectively. In addition to E-cad loss, our approach makes it possible to distinguish the proportions of hybrid E/M cancer cells exhibiting the different combinations of weak vs. strong pan-CK expression and weak vs. strong VIM expression. However, these distinctions need to determine thresholds on IHC staining intensity that could be more difficult to reproduce in clinical practice.

## 5. Conclusions

In conclusion, we propose in the present study, a new method able to detect and quantify cancer cells with a hybrid E/M state, in large series of paraffin-embedded tissue samples. By applying it to one hundred urothelial carcinomas, we showed that the presence of at least a small amount of these hybrid cancer cells is associated with tumor aggressiveness (high stage, high grade, divergent differentiation, N+, and CIS) and poor prognosis (regarding DFS and OS). Although validation on a larger case series is required, our data support the hybrid E/M score as a promising prognostic biomarker for urothelial cancer patients. We are now applying the same methodology to other cancers where EMT is involved.

## Figures and Tables

**Table 1:** Relations between the presence of hybrid E/M cancer cells and clinicopathological variables.

Variable	Category	E/M score > 2%		*p*-Value ^**1**^
**T**	Ta–T1 (*n* = 48)	6	(13%)	<10^−6^
	T2–T4 (*n* = 65)	38	(58%)	
**N**	N0 (*n* = 92)	32	(35%)	0.08
	N+ (*n* = 31)	12	(57%)	
**M**	M0 (*n* = 108)	41	(38%)	0.38
	M1 (*n* = 5)	3	(60%)	
**Stage**	0–I (*n* = 48)	6	(13%)	<10^−6^
	II–IV (*n* = 65)	38	(58%)	
**Grade**	low (*n* = 25)	1	(4%)	0.00002
	high (*n* = 88)	43	(49%)	
**Divergent differentiation**	no (*n* = 88)	29	(33%)	0.02
	yes (*n* = 25)	15	(60%)	
**CIS** ^**2**^	no (*n* = 83)	27	(33%)	0.03
	yes (*n* = 30)	17	(57%)	
**Multifocality**	no (*n* = 91)	40	(44%)	0.03
	yes (*n* = 22)	4	(18%)	
**LVI** ^**3**^	no (*n* = 72)	24	(33%)	0.11
	yes (*n* = 41)	20	(49%)	

^1^ Two-tailed Fisher’s exact test; ^2^ concomitant in situ carcinoma; ^3^ lymphovascular invasion.

**Table 2:** Cox regression model for disease-free survival. The model includes the most contributive clinicopathological variables (see Appendix Table A1) and the binarized E/M score.

Risk Factor	Beta	SE ^1^	HR ^2^	95%	CI ^3^	*p*-Value
N+	1.4426	0.4025	4.2316	1.9226	9.3138	0.0003
CIS (yes)	0.8440	0.3639	2.3256	1.1397	4.7457	0.0204
E/M > 0.02	0.9699	0.3696	2.6376	1.2782	5.4429	0.0087

^1^ Standard error; ^2^ hazard ratio; ^3^ 95% confidence interval for HR.

**Table 3:** Cox regression model for disease-free survival. The model includes the most contributive clinicopathological variables (see Appendix Table A1) and the binarized E/M score.

Risk Factor	Beta	SE ^1^	HR ^2^	95%	CI ^3^	*p*-Value
N+	1.4961	0.3969	4.4640	2.0544	9.7000	0.0002
CIS (yes)	0.9147	0.3570	2.4960	1.2398	5.0249	0.0104
Advanced E/M > 0.01	1.0993	0.3747	3.0019	1.4402	6.2572	0.0034

^1^ Standard error; ^2^ hazard ratio; ^3^ 95% confidence interval for HR.

## Appendix A

**Table A1:** Cox regression model for disease-free survival. The model includes the clinicopathological variables selected using univariate survival analysis (with *p* < 0.10).

Risk Factor	Beta	SE ^1^	HR ^2^	95%	CI ^3^	*p*-Value
Differentiation (yes)	0.6342	0.4145	1.8855	0.8367	4.2491	0.126
High grade	0.2819	0.5265	1.3256	0.4724	3.7201	0.592
T2-T4	0.1084	0.2644	1.1145	0.6638	1.8710	0.682
N+	1.2436	0.5496	3.4680	1.1809	10.1843	0.024
CIS (yes)	0.7873	0.3819	2.1974	1.0395	4.6448	0.039
LVI (yes)	0.1393	0.6517	1.1494	0.3204	4.1233	0.831

^1^ Standard error; ^2^ hazard ratio; ^3^ 95% confidence interval for HR.
